# PERK interacts with FLNA to regulate ER-PM contact sites

**DOI:** 10.18632/oncotarget.22384

**Published:** 2017-11-11

**Authors:** Alexander R. van Vliet, Patrizia Agostinis

**Affiliations:** Patrizia Agostinis: Department Cellular and Molecular Medicine, Laboratory of Cell Death and Therapy, KU Leuven, Leuven, Belgium

**Keywords:** PERK, unfolded protein response, endoplasmic reticulum stress, ER-PM contact sites, calcium signalling

The endoplasmic reticulum (ER) is a major organelle of the cell and represents the bulk of cellular protein folding. Proteins folded in the ER are destined for secretion or plasma membrane (PM) incorporation. To ensure proper folding, the ER contains chaperones assisting the folding process. However, in certain physiological conditions, this folding machinery is overwhelmed and can no longer cope with the cell’s demands, a condition termed ER stress. Three pathways have evolved that sense and ameliorate ER stress, or when this fails, induce cell death. Together they are termed the unfolded protein response (UPR) [1]. The UPR consists of the kinase Inositol Requiring Enzyme 1 (IRE1), activating transcription factor 6 (ATF6) and the protein kinase RNA-like endoplasmic reticulum kinase (PERK). One of the main mechanisms of UPR-mediated ER stress relief comes in the form of PERK-mediated phosphorylation of eukaryotic Initiation Factor 2 α (eIF2α), causing translational arrest [1]. Besides its canonical role of splicing XBP-1, IRE1 possesses an mRNA splicing activity, termed regulated IRE1-dependent decay and a scaffolding role that functions as a signalling platform for TRAF2 and JNK1 kinase [2]. In contrast, PERK had mainly been shown to phosphorylate its two canonical substrates, NRF2 and eIF2α [1]. We previously discovered that PERK plays a role in ER-mitochondria contact sites, contributing to the tethering of ER and mitochondria [3]. As a bridge between these organelles, PERK plays a role in cell death, by favouring the transfer of pro-apoptotic factors from the ER to the mitochondria [3], and could regulate inflammation and autophagy, processes known to be regulated at the ER-mitochondria contact sites [4]. It had also been shown that PERK plays a role in immunogenic cell death (ICD). Interestingly in ICD, PERK enacts its signalling role independently of eIF2α phosphorylation, relying instead on Ca^2+^ signals and actin-cytoskeleton mediated exocytosis of danger signals [5]. PERK is also involved in intracellular Ca^2+^ fluxes and store-operated Ca^2+^ entry (SOCE), a key process in restoring intracellular Ca^2+^ levels [6]. Intriguingly, the mechanisms through which PERK enacted these roles remained largely unknown, and the notion that PERK could function as a scaffold, similarly to IRE1, remained unexplored. In new research, we wanted to investigate this hypothesis. Utilizing proximity-dependent biotin identification (BioID), we identified a novel PERK interacting protein in the F-actin cytoskeletal protein FLNA. FLNA, a cytosolic protein consisting of 24 β-barrel domains, an F-actin binding domain and a dimerization domain forms cross-linked, orthogonal networks of F-Actin. FLNA binds to PERK through its C-terminus and connects this ER stress kinase to the F-actin cytoskeleton. Upon deletion of either PERK or FLNA, the F-actin cytoskeleton is modified from a homogenous to a cortical distribution, becoming enriched at the PM. We hypothesized that this excessive relocalization of F-actin beneath the PM could lead to a defect in ER-PM contact site formation in terms of SOCE, possibly uncovering a mechanism important in previous studies. Upon ER Ca^2+^ depletion, the ER localized Stromal interaction molecule 1 (STIM1) multimerizes and becomes active, translocating to the PM to interact with the PM-localized Ca^2+^ channel Orai1 [6]. This interaction causes the influx of extracellular Ca^2+^ through SOCE. Interestingly, we found that STIM1 migration to the PM itself was significantly diminished. To investigate whether this phenotype was unique to STIM1 or was a general ER-PM contact site defect, we looked at another ER resident protein, Extended Synaptotagmin-1 (E-Syt1). E-Syt1 translocates to the PM upon cytosolic Ca^2+^ increase, sensed by its C2 domains, to form a direct ER-PM tether and has been previously linked with STIM1 [7]. Indeed, E-Syt1 also exhibited decreased PM localization upon ER Ca^2+^ depletion after loss of PERK. Expressing a kinase dead mutant of PERK rescued both STIM1 and E-Syt1 mediated ER-PM contact site formation, indicating the redundancy of PERK’s kinase function for this effect. Highlighting the importance of F-actin in modulating ER-PM contact site formation, the addition of F-actin polarizing/depolarizing agents could abolish or rescue the decrease in E-Syt1 and STIM1 relocalization, respectively. Further investigating this link, we found that STIM1 and E-Syt1 puncta formed in an organized manner along F-actin fibers present directly beneath the PM. This tight connection was strongly diminished upon loss of PERK.

PERK activation during the UPR is caused by releasing the inhibitory action of BiP on PERK’s luminal domain when unfolded proteins accumulate in the ER [1]. Intriguingly, we show that PERK can be activated through cytosolic Ca^2+^ increases caused by ER Ca^2+^ depletion and that this event leads to PERK dimerization and increased FLNA binding. This is independent of PERK’s luminal domain. We found that the dimerization event of PERK is a key part of its mode of action, since artificially inducing PERK dimerization, with the GSK inhibitor GSK2606414, increased FLNA interaction and led to increased STIM1 PM recruitment when followed by ER Ca^2+^ depletion. This showed that the PERK-FLNA-F-actin axis, regulating ER-PM contact site formation after ER Ca^2+^ depletion does not require PERK’s phosphorylation activity but relies on its dimerization.

Researchers aiming at harnessing PERK in clinical settings have tried to modulate its kinase function to abolish its function. However, this study shows that this aim may have other unintended effects. ER-PM contact sites have many important cellular functions, not limited to SOCE and Ca^2+^ signalling, but also include lipid transfer, regulating PM lipid composition and endo- and exocytosis. The finding that PERK could play a role in the formation of ER-PM contacts could thus open new doors [8].
